# Evaluating the feasibility of cognitive impairment detection in Alzheimer’s disease screening using a computerized visual dynamic test

**DOI:** 10.1186/s12984-023-01155-2

**Published:** 2023-04-12

**Authors:** Eduardo Perez-Valero, Christian A. Morillas Gutierrez, Miguel Angel Lopez-Gordo, Samuel López Alcalde

**Affiliations:** 1 Department of Computer Engineering, Automation and Robotics, University of Granada, Granada, Spain; 2 Department of Signal Theory, Telematics, and Communications, University of Granada, Granada, Spain; 3 Brain-Computer Interfaces Laboratory, Research Centre for Information and Communications Technologies, Granada, Spain; 4 Hospital Universitario San Rafael, Granada, Spain

**Keywords:** Alzheimer’s disease, Screening, Visual dynamics, Cognitive tests

## Abstract

**Background:**

Alzheimer’s disease (AD) is a neurodegenerative disease without known cure. However, early medical treatment can help control its progression and postpone intellectual decay. Since AD is preceded by a period of cognitive deterioration, the effective assessment of cognitive capabilities is crucial to develop reliable screening procedures. For this purpose, cognitive tests are extensively used to evaluate cognitive areas such as language, attention, or memory.

**Methods:**

In this work, we analyzed the potential of a visual dynamics evaluation, the rapid serial visual presentation task (RSVP), for the detection of cognitive impairment in AD. We compared this evaluation with two of the most extended brief cognitive tests applied in Spain: the Clock-drawing test (CDT) and the Phototest. For this purpose, we assessed a group of patients (mild AD and mild cognitive impairment) and controls, and we evaluated the ability of the three tests for the discrimination of the two groups.

**Results:**

The preliminary results obtained suggest the RSVP performance is statistically higher for the controls than for the patients (p-value = 0.013). Furthermore, we obtained promising classification results for this test (mean accuracy of 0.91 with 95% confidence interval 0.72, 0.97).

**Conclusions:**

Since the RSVP is a computerized, auto-scored, and potentially self-administered brief test, it could contribute to speeding-up cognitive impairment screening and to reducing the associated costs. Furthermore, this evaluation could be combined with other tests to augment the efficiency of cognitive impairment screening protocols and to potentially monitor patients under medical treatment.

## Background

Dementia is an umbrella term used to describe the loss of cognitive functioning that affects individuals to the extent of interfering with daily-life activities [1]. Presently, around fifty million people live with dementia and the prevalence is expected to almost triple by 2050 owing to the aging of the global population [2]. Among the diseases that cause dementia, Alzheimer’s disease (AD) is the most common, since it represents between 60% and 80% of the cases [2]. AD is a neurodegenerative disease that affects multiple cognitive areas such as memory, orientation, or language [3]. Although the first case of AD was reported in 1901, its etiology still remains undetermined. Nonetheless, researchers have identified two main hallmarks linked to AD: amyloid plaques and neurofibrillary tangles [4]. The former are protein deposits that lose their standard structure and accumulate around the neurons, whilst the latter are thickened fibrils surrounding their nucleus. Both structures damage the neuronal processes and start to form more than ten years before the impairment is notable. On the other hand, mild cognitive impairment (MCI) refers to a transitional stage between normal aging and AD [5]. MCI patients experience minor memory losses which do not interfere with daily-life activities. However, they transition to AD faster than healthy individuals of the same age. In this context, although there is no cure for AD, medical treatment can contribute to controlling the progression of the disease and to delaying cognitive decline [6]. In this context, early detection is crucial for the wellness expectations of the patients.

With this in mind, primary healthcare represents the front-line for the detection of cognitive impairment before more complex procedures such as magnetic resonance imaging [7], positron emission tomography [8], or cerebro-spinal fluid analysis [9] are conducted. In this respect, cognitive tests have been extensively used to detect cognitive impairment via the assessment of the cognitive areas affected earlier in the course of AD, such as visuo-spatial ability, verbal fluency, and episodic memory [10–13]. Typically, cognitive tests are incorporated into test batteries in order to evaluate multiple cognitive areas in a single session [14, 15]. The most popular test batteries are the mini-mental state examination [16] and the Montreal cognitive assessment [17], although other evaluations such as the Test your memory assessment [18] and the Mini-Cog [19] have been proposed.

**Fig. 1:**
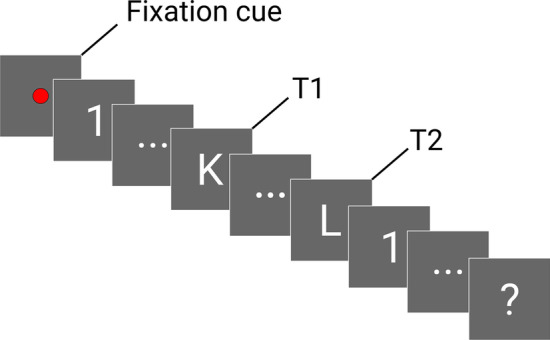
Diagram of a trial from the rapid serial visual presentation. The squares represent the stimuli presented during the trial: distractors (numbers) and targets (letters). The “...” character between T1 and T2 represents the intervening distractors. The “?” character represents the reporting stage

In this connection, traditional cognitive tests like the Clock-drawing test (CDT) [20], the animal naming test [21], or the abbreviated mental test [22], focus mainly on memory and executive functioning. Therefore, other cognitive areas affected early in the AD course, such as visual processing, may be overlooked. For the past decade, multiple works have reported reduced performance of AD patients in cognitive tests involving visual processing. For instance, [23] applied the theory of visual attention to the results of a letter-identification paradigm and found that visual impairments follow an orderly progression along the AD course. Similarly, [24] proposed the integrated cognitive assessment, a test for the identification of animal versus non-animal images, as a reliable tool for cognitive impairment screening in dementia. Alternatively, [25], and [26] found deficits in the visual processing capabilities of AD patients compared to healthy age-matched controls when they were evaluated using the rapid serial visual presentation (RSVP). In this task, the patients are required to identify two target letters separated by a number of intervening distractors which are rapidly presented on the computer screen (see Fig. 1). Trials with different number of intervening distractors are designed to evaluate the attentional and visual dynamics capabilities of the patients.

In this paper, our goal is to evaluate the feasibility of a computerized visual dynamics test for the detection of cognitive impairment in AD screening. To this end, we implemented a version of the RSVP and we conducted a preliminary study to evaluate the performance of two groups: patients (mild AD and MCI-non-AD) and healthy age-matched controls. We evaluated the performance of the participants in the RSVP in terms of the so-called attentional blink (AB) and attentional masking (AM). AB refers to the inability to recall T2 after correctly reporting T1. On the other hand, AM refers to the inability to recall T1 after correctly reporting T2. According to previous studies, healthy older adults do not show the latter effect [25, 26]. Considering this, our motivation to study this test was two-fold: (1) the RSVP assesses visual dynamics and working memory, two of the first areas affected by AD [24]; and (2), the RSVP is an auto-scored and computerized test. With this in mind, we evaluated the ability of the RSVP to discriminate the two groups studied, and we compared the results with two of the most popular brief cognitive tests used in Spain for AD screening: the CDT and the Phototest. We conducted this preliminary study in collaboration with the cognitive and behavioral neurology unit (CBNU) at Hospital Universitario Virgen de las Nieves de Granada (Spain).

## Methods

In this section, we report the demographic details of the participants engaged, the implementation of the cognitive tests evaluated, and the analysis of the results obtained in this preliminary study.

### Participants

Members from the CBNU recruited twenty-five participants for this study and split them into two groups: cognitive impairment (CI) and healthy controls (HC). The CI group included mild AD and MCI-non-AD patients, whilst the HC group included age-matched healthy controls who did not suffer from any cognitive disease and presented normal to corrected vision. The exclusion criteria applied to the participants included: receiving a medical treatment which could alter cognitive performance, suffering from visual/auditory affections that could prevent the participants from completing the cognitive tests, and, in the case of the CI group, the presence of a neurological disorder aside from mild AD/MCI-non-AD. Following these criteria, we discarded the data from three participants from the original sample. Therefore, the sample analyzed in this study included 22 participants: the CI group included 13 participants (3 females, mean age 70.9 ± 6.0) with 8 mild AD and 5 MCI-non-AD, whilst HC group included 9 age-matched healthy participants (8 females, mean age 66.7 ± 3.4). The participants in the CI group were patients of the CBNU at Hospital Universitario Virgen de las Nieves who were diagnosed through one of the following medical procedures during the year prior to the start of this study:

- CSF analysis. This analysis was performed by two different laboratories during the recruitment period. The reference cutoff value for the patients was that stipulated by the laboratory based on a model of non-AD patients versus AD patients without age stratification. CSF was acquired through lumbar puncture using a syringe and a 20-gauge needle. Samples were collected in polypropylene collection tubes and instantly sent to the laboratory, where an ELISA Innotest assay was utilized to determine the levels of A$\beta$42, total-$\tau$, and $\tau$-phosphorylated fraction. The results of the analysis were codified as normal or pathological.
- PET scan. PET-Amyloid was analyzed using 18F-florbetaben (FBB) by qualified nuclear medicine specialists who had completed the learning curve for accredited PET-FBB scan interpretation and were blinded to the clinical situation of the patients. They evaluated the presence of amyloid plaques, and consequently reported the scan results as positive (loss of gray-white matter contrast; regional cortical tracer uptake in any cortical target region: lateral temporal, frontal, posterior cingulate precuneus, or parietal), or negative (good gray-white matter contrast; no tracer uptake in target regions). The cases with doubtful results were examined by both specialists to achieve an agreement.

The neurologists at the CBNU, who collaborated in this preliminary study, performed a clinical diagnosis based on the results of the aforementioned clinical trials. Consequently, they labeled the patients as mild AD (CSF pathological results or PET positive amyloid plaque presence) and MCI-non-AD (CSF normal results or PET negative amyloid plaque presence). The study was conducted according to a protocol authorized by the ethics committee at Hospital Universitario Virgen de las Nieves de Granada. In addition, all the participants signed an informed consent prior to the onset of the experiment, and they were monitored by clinical personnel throughout the entire experiment session.

### Cognitive tests

In this subsection, we report the methodological details regarding the three cognitive tests analyzed in this preliminary study: the CDT, the Phototest, and the RSVP.

#### The clock-drawing test

The CDT is one of the most widespread brief cognitive tests for the detection of dementia worldwide owing to its simplicity and reduced duration of approximately 2 min [27–29]. This test is designed to evaluate visuo-spatial capabilities and executive functioning. For this purpose, the patients are asked to draw a clock, including clock face, numbers, and clock hands indicating a particular time. Notably, the CDT relies on the grapho-motor abilities of the patients and manual correction, what hinders the implementation of a computerized version. For this study, we used the CDT version scored from 0 to 7 [19, 20].

#### The Phototest

The Phototest is one of the most studied brief cognitive test for cognitive impairment detection in Spain [30, 31]. This test evaluates episodic memory, executive functioning, and verbal fluency. For this purpose, the patients are required to recall six objects previously identified from an illustration. Additionally, between the naming task and the recall task, the patients are asked to evoke male and female names. The use of the Phototest for the detection of cognitive impairment and dementia has been extensively validated in the literature [19, 32, 33]. The main advantages of the Phototest are its reduced duration of less than three minutes and its robustness regarding educational level and illiteracy. To prevent the patients from learning the objects in the illustration, there are multiple versions of the test, each including different objects [34]. For this study, we used the version including a card deck, a car, a pear, a trumpet, a pair of shoes, and a spoon.

#### The rapid serial visual presentation (RSVP)

The RSVP represents a valid framework for the assessment of visual attention and visual dynamics. For this purpose, the participants are presented a rapid stream of characters including multiple numbers and two letters. The goal of the task is to identify the two letters, T1 and T2, in each trial. Inspired by two previous works that studied the RSVP in AD patients [25, 26], we implemented this paradigm using PsychoPy 2020.1 Python toolbox according to the guidelines outlined subsequently. For the targets, we considered all the letters in the Latin alphabet with the exception of I, O, Q, S, U, V, W, X, and Z, since they could be easily confused with numbers or between them. For similar reasons, we only considered the following numbers: 3, 4, 6, 7, 8, and 9. To present the stream of characters we used a laptop with a screen refresh rate of 60 Hz, hence, each character was presented for 150 ms (9 frames) with no blank screen intervals between consecutive characters. Both the numbers and the letters were displayed in white Arial font on a dark background using a font size large enough to be identified by the participants. We implemented seven type of trials according to the number of intervening distractors placed between T1 and T2. These correspond to separations of 0 (no distractors between T1 and T2), 1, 2, 4, 5, 6, and 8. The test consisted of 70 trials (10 per separation). Each trial began with a red fixation cue followed by a tone beep that prompted the participants to focus on the screen. At the end of each trial, the participants were asked to verbally report the targets, and a technician marked the responses using a slider displayed in the RSVP software. Figure 1 represents the structure of a trial of the RSVP implemented for this study.

### Statistical analysis

First, we compared the performance of the two groups on the three tests analyzed in this work. For the CDT and the Phototest, we determined the participant performance as the score obtained for each corresponding test. Alternatively, for the RSVP, we quantified performance using two global metrics: global AB and global AM. We calculated these metrics as the average of the AB and the AM for the trials with 0, 1, and 2 intervening distractors. For this purpose, we estimated AB and AM as $P(T2 \mid T1)$ and $P(T2 \mid T1)$, respectively. After estimating the performance of the two groups on the three tests, we applied the non-parametric Mann–Whitney U test to compare the distributions of the two groups. We applied this test because it represents a more conservative approach than parametric alternatives, especially when the sample size is reduced. For all the hypothesis tests we set the significance level at 5%. For the statistical analysis of each cognitive test, we estimated the average score obtained for the two groups, the U-value, the p-value, and the effect size. Particularly, we estimated the common language effect size, which was first introduced in [35], and represents the probability that a score selected randomly from the distribution of the first group will be greater than a score selected from the distribution of the other group.

### Classification analysis

On the other hand, we also performed a classification analysis. To do so, we evaluated the performance of the three tests studied in this work to discriminate the two groups. For the CDT and the Phototest, we applied the corresponding performance thresholds established in the literature for the detection of cognitive impairment (6 and 29 points, respectively) [30, 36]. Consequently, we classified the participants as CI if their performance was below the threshold, and as HC otherwise. For the RSVP, since there are no established performance thresholds in literature, we evaluated two classification approaches:

- Threshold approach. After visual inspection of the RSVP results, we attempted a threshold-based classification using global AM. We selected this metric because it represents the area of the performance curves where the differences between the two groups are more pronounced. We evaluated this approach through leave-one-out cross-validation. Consequently, for each iteration, the training set was created using the data corresponding to all the participants but one, whose data was reserved for the holdout (test) set. In each iteration, we determined the optimal performance threshold using the training set, and we used that threshold to predict the holdout. We refer to this approach as RSVP (AM) throughout the rest of this paper.
- Logistic regression approach. In this case, we created a feature matrix whose rows corresponded to the participants, and whose columns corresponded to the AB and AM performance for all the different trials performed. Consequently, the dimension of the feature matrix was 22 × 14. To estimate the hyperparameters of the logistic regression model we applied grid-search, and to evaluate its performance, we applied cross-validation following a leave-one-out strategy, as we did for the RSVP (AM) approach. We refer to this approach as RSVP (LR) throughout the rest of this paper.

**Fig. 2:**
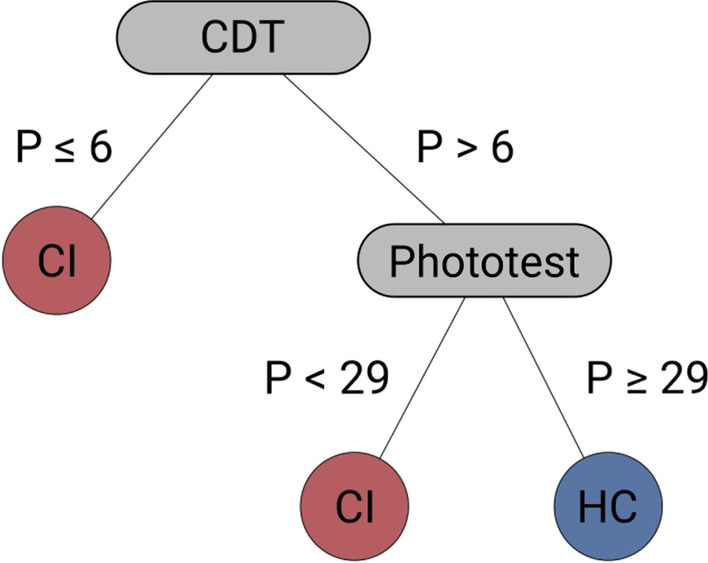
Sequential analysis. First, the CDT score is considered. If this score is below the established CDT threshold for cognitive impairment, the participants are identified as CI; otherwise, the Phototest score is considered; if this score is below the established Phototest threshold for cognitive impairment, the participants are identified as CI; otherwise, they are identified as HC

In addition, we also estimated the classification performance of a sequential analysis approach using the CDT and the Phototest. This approach mimics the examination potentially performed by neuro-psychologists during cognitive impairment screening. First, the CDT is considered: if the scores are below the CDT performance threshold, the participants are classified as CI, otherwise, the Phototest is considered; if the scores are below the Phototest performance threshold, the participants are classified as CI, otherwise, they are classified as HC. Figure 2 represents the flow of the sequential analysis. To evaluate the classification performance of the tests, we estimated precision, recall, accuracy, and the confusion matrices. For the two RSVP approaches, the reported accuracy refers to the average value obtained via cross-validation.

## Results

In this section, we report the results of the statistical analysis performed to compare the test results of the two groups and the classification analysis carried out to discriminate the participants.

### Statistical analysis

**Fig. 3:**
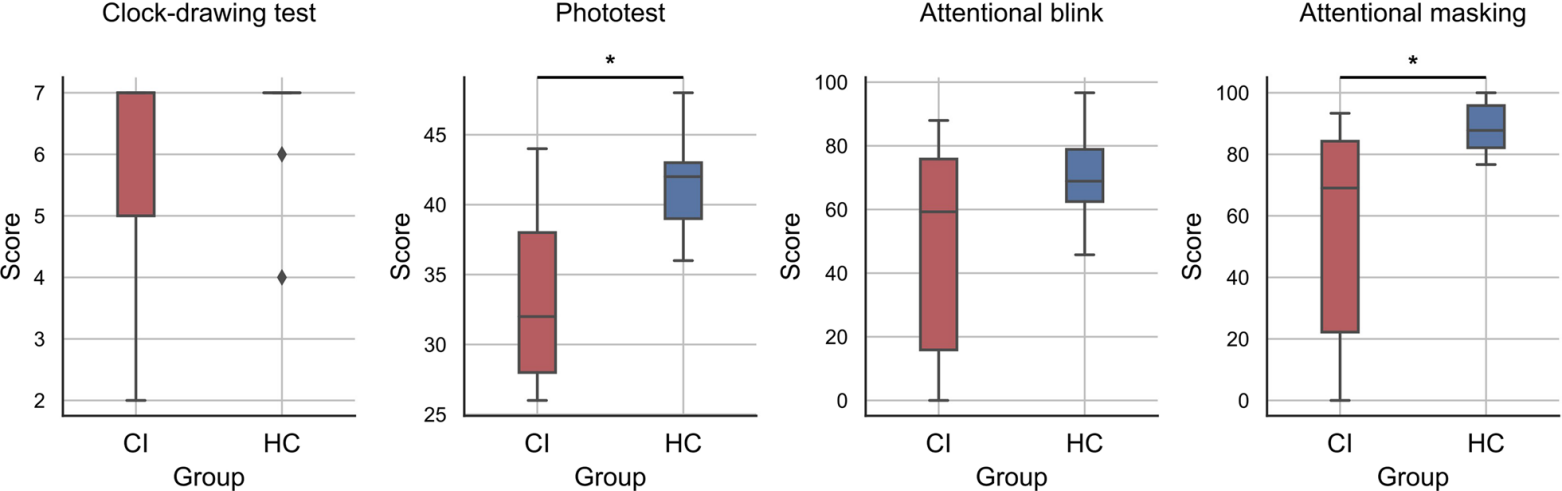
Group distributions for the results of the CDT, the Phototest, and the RSVP. The distributions are represented through a boxplot. Asterisks indicate p-value $\le 0.05$

**Fig. 4:**
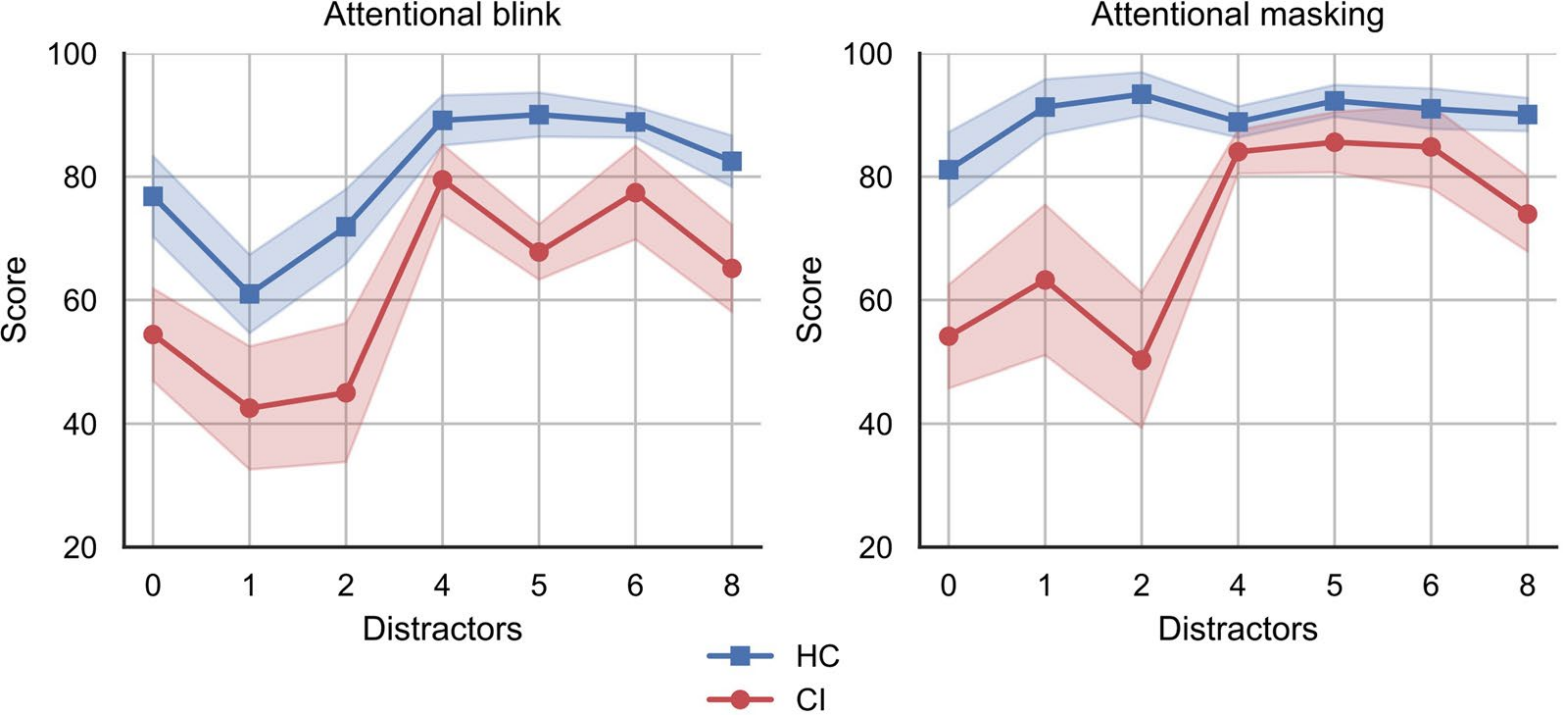
RSVP performance by group in terms of attentional blink and attentional masking. The X axis represents the different type of trials conducted in the RSVP according to the number of intervening distractors. For the attentional blink plot, the Y axis represents $P(T2 \mid T1)$, whilst for the attentional masking it represents $P(T1 \mid T2)$. The shades represent the standard error of the mean

Figure 3 represents the distributions of the results obtained by the two groups (CI and HC) for the CDT, the Phototest, and the RSVP (global AB and global AM). As evidenced in this figure, only two participants in the HC group did not reach the highest possible score in the CDT. Nonetheless, the performance of these two participants can be considered as an outlier, since their results were more than 1.5 standard deviations below the first quartile. We have reported the details of these statistical comparisons in Table 1. Among all the comparisons, only the Phototest and the RSVP (global AM) yielded statistically significant differences between the two groups (p-values of 0.005 and 0.013, respectively). Alternatively, Fig. 4 shows the results obtained by the two groups in the RSVP test in terms of AB and AM for the different number of intervening distractors employed during the task.

### Classification analysis

Table 2 represents the classification report for the three tests analyzed in this preliminary study and for the sequential approach described in subsection Classification analysis of the Methods section. The precision and recall metrics estimated in the table are the average of the values obtained for each group (CI and HC). Furthermore, Table 3 shows the confusion matrices obtained from the classification analysis of the three tests. Lastly, for the sake of reproducibility, Table 4 shows the range of the hyperparameters that we optimized through grid-search cross-validation along with the best combination found.

**Table 1:** Statistical comparison between the two groups (CI and HC) for the different cognitive tests evaluated

Test	Mean (CI)	Mean (HC)	p-value	U-value	Effect size
CDT	5.69 ± 0.49	6.56 ± 0.32	0.229	42.5	0.64
Phototest	33.08 ± 1.63	41.56 ± 1.07	**0.005**	16.0	0.86
RSVP (global AM)	47.32 ± 8.77	69.89 ± 4.68	**0.013**	21.0	0.82
RSVP (global AB)	55.88 ± 8.77	88.61 ± 2.62	0.229	40.0	0.66

The columns indicate, from left to right, the cognitive test, the mean score ± the standard error of the mean for the CI and the HC groups, the p-value, the U-value, and the effect size. In bold, statistically significant p-values

**Table 2:** Performance of the tests evaluated in this work for the classification of the two groups (CI vs HC)

Test	Precision	Recall	Accuracy
CDT	0.62	0.62	0.59 [0.39, 0.77]
Phototest	0.76	0.69	0.64 [0.43, 0.80]
Sequential	0.66	0.66	0.64 [0.43, 0.80]
RSVP (AM)	0.72	0.71	0.68 [0.47, 0.84]
RSVP (LR)	0.91	0.91	0.91 [0.72, 0.97]

The numbers in brackets next to the accuracy represent the 95% confidence intervals around the mean considering the predictions as a series of Bernoulli trials

**Table 3:** Confusion matrices obtained after using the three tests studied to classify the two groups (CI vs HC)

	CDT		Phototest		Sequential		RSVP (AM)		RSVP (LR)	
	CI	HC	CI	HC	CI	HC	CI	HC	CI	HC
PAT	**6**	7	**5**	8	**7**	6	**7**	6	**12**	1
HC	2	**7**	0	**9**	2	**7**	1	**8**	1	**8**

The bold cells denote the true positives and true negatives

**Table 4:** Ranges of the logistic regression hyperparameters optimized through grid-search

Hyperparameter	Range	Best value
penalty	l1, l2	l2
C	$10^{-7}, 10^{-6}, ..., 10^7$	$10^5$

The right-most column indicates the best value found using cross-validation. C refers to the inverse of the regularization strength. For a detailed description of the parameters, refer to the scikit-learn documentation [51]

## Discussion

The goal of this work was to evaluate the feasibility of the RSVP to detect cognitive impairment in the context of AD screening. For this purpose, we evaluated two groups of participants (mild AD/MCI-non-AD patients and controls) on the RSVP, the CDT, and the Phototest. The CDT (drawing) and the Phototest (evoking and recalling) demand different actions from the participants compared to the RSVP (identifying visual targets) and also involve different cognitive areas. However, these cognitive tests have been individually evaluated in the literature for cognitive impairment detection, and they are designed to assess mental areas frequently affected early in the course of the cognitive impairment. Furthermore, the CDT and the Phototest are two of the most widely applied brief cognitive tests in Spain, what also motivated our selection with a view to perform a preliminary comparison with the RSVP. To compare the results of the CDT, the Phototest, and the RSVP, we analyzed the statistical differences in terms of performance between the two groups, and we evaluated the classification performance of the cognitive tests for their discrimination. The preliminary results that we obtained suggest that the RSVP may represent a valuable alternative for the detection of cognitive impairment in AD screening.

Regarding the statistical results presented in Table 1, the average performance of the HC group was superior to the CI group for all the cognitive tests analyzed. According to Fig. 3, all the participants in the HC group except two obtained the highest score in the CDT. Alternatively, 7 out of 13 participants in the CI group also obtained the highest score. This suggests that the CDT may have reduced sensitivity for the detection of cognitive impairment in mild AD and MCI-non-AD patients, which agrees with the prevalent view in the literature [30, 37]. Indeed, we did not find a statistically significant difference between the two groups for this test. With respect to the Phototest, we found a statistically significant difference in the performance of the two groups (p-value = 0.005 and 0.86 effect size). This was expected since the Phototest has been extensively validated for the detection of cognitive impairment in dementia and mild dementia [32, 38]. With respect to the RSVP, we found a statistically significant difference in the performance of the two groups for the global AM metric (p-value = 0.013 and 0.82 effect size), what agrees with previous studies in the literature where the RSVP was evaluated in AD patients. These studies showed that the patients are vulnerable to AM as opposed to healthy controls [25, 26]. Such conclusions stem from the deficits in temporal dynamics of visual perception observed in AD patients, what causes the arrival of the second stimulus (T2) to block recall of the first stimulus (T1), and they are consistent with the results presented in Fig. 4. Noteworthy, in [25, 26], the study groups only included AD patients. In contrast, we also included MCI-non-AD patients following a strategy similar to [38]. However, the results reported in Figs. 3 and 4 suggest that MCI-non-AD patients may also experience AB and AM. This could be explained through the deficits in visual processing speed shown by MCI patients [24, 39]. Considering this, the preliminary results that we obtained for the RSVP are in line with preceding studies which have associated deficits in visual function with AD [40, 41]. Furthermore, the RSVP involves the visual cortex and working memory, both potentially affected even before AD symptoms emerge. Alternatively, our preliminary results are also consistent with recent studies linking MCI and reduced visual processing [39, 42, 43]. These deficits in MCI and AD patients have been evidenced also in previous works analyzing brain electrical activity [44–46].

In terms of the ability of the tests to discriminate the participants of the two groups, Table 3 hints a disparity in classification performance across tests. As anticipated by the statistical analysis, the CDT miss-classified 7 out of 13 participants from the CI group as HC. Again, this could be explained by the simplicity of the test (the proposed task may not be challenging enough for mild AD and MCI-non-AD patients). With respect to the Phototest, although this evaluation correctly identified all the controls, it missclassified 8 out of 13 participants from the CI group. These results could be associated with the small size of the sample considered in this preliminary study. The sequential application of the CDT and the Phototest slightly improved the results compared to using only the CDT, but did not improve the overall results compared to using only the Phototest. On the other hand, the two RSVP approaches evaluated via cross-validation yielded better classification results. The threshold-based approach using global AM returned slightly better results compared to the sequential approach (one additional participant from the HC group was correctly classified). Lastly, the logistic regression approach yielded the best classification results as only one of the participants from each group was miss-classified.

Models previously presented have associated the reduced RSVP performance observed in AD patients with impaired working memory. In AB, the processing of the second stimulus may be undermined because the processing mechanisms are engaged with the first stimulus. On the other hand, AM may be a result of diminished consolidation of the first stimulus [25, 26]. The preliminary results that we obtained suggest the potential capacity of visuo-spatial capabilities and executive functioning to detect cognitive impairment in AD screening.

With respect to implementation features, the cognitive tests analyzed in this study present advantages and drawbacks. In terms of administration, the CDT and the Phototest require a clinical professional to be conducted and scored, since these tests involve drawing and evoking, respectively. Conversely, although for this preliminary study we asked the participants to verbally report the answers, the RSVP is computerized and auto-scored. Hence this test could be easily self-administered assuming basic computer skills through a keyboard or a touch-screen. With respect to duration, the CDT and the Phototest are very brief (2 and 3 min, respectively), whilst the RSVP is the longest among the tests evaluated in this study (10 min). Finally, in terms of patient requirements, the CDT demands minimal graphical skills and numerical knowledge, the Phototest only requires the participants to identify daily-life images and evoke names, and the RSVP requires healthy visual perception and, in case of self-administration, basic computer skills. Considering this, only the Phototest has been already validated for the detection of cognitive impairment in the illiterate [36]. Whilst the features described in this paragraph did not impact the analysis presented in this preliminary study, because all the participants analyzed were able to undertake the three cognitive tests, if we consider the potential implementation of the tests as an online service, which seems to be the course to follow in dementia screening [47–50], the RSVP may represent a more appropriate candidate based on its computerized and auto-scored nature. This implementation could potentially allow the users to access the test from home, with the assistance of their caregivers if needed. As a result, this may contribute to relieving the congestion in primary healthcare, to reducing the associated costs, and to supporting the creation of longitudinal databases. Nonetheless, further research is required before this kind of approaches are transferred to the clinical ecosystem.

Finally, although the results presented in this paper are promising (as evidenced, for instance, by the large effect size obtained for the RSVP global AM metric), this preliminary study presents some limitations. First, to render a comprehensive assessment of the proposed evaluation, different age and educational level groups have to be evaluated individually. This would enable, for example, the assessment of the feasibility of the RSVP to evaluate patients with lower educational level, which is crucial for this kind of evaluations. Furthermore, in this study we did not analyze the potential differences between sex because the participant sample was reduced and not balanced. Although we do not anticipate such differences, further studies are required to validate this hypothesis on a larger participant sample. In addition to age, educational level, and sex, the proposed evaluation must be analyzed for the different phases of dementia in order to uncover potential biases resulting from the sample grouping examined in this preliminary study (which included mild AD and MCI-non-AD patients). Lastly, we did not assess the self-administration capability of the RSVP. Additional works must validate this in order to evaluate the feasibility of implementing the RSVP as an independent online service for assisting the detection of cognitive impairment.

## Conclusions

In this work, we evaluated the feasibility of the RSVP, a visual dynamics test, for the detection of cognitive impairment in the context of AD screening. For this purpose, we conducted a preliminary study involving 13 patients (mild AD and MCI-non-AD) and 9 age-matched healthy controls. We used cerebrospinal fluid analysis and positron emission tomography scans as the gold standard for the diagnosis of the patients. We evaluated the participants using the RSVP and two of the brief cognitive tests most widely used in Spain for AD screening: the Clock-drawing test and the Phototest. Then, we statistically compared the results of the two groups and we evaluated the classification capabilities of the three tests. For the classification analysis, we evaluated the CDT and the Phototest (individually and sequentially combined) using the thresholds reported in the literature. We also evaluated the RSVP via cross-validation using a threshold-based approach and a logistic regression model. The logistic regression yielded the best results in terms of classification accuracy (0.91). These preliminary results suggest that the evaluation of visual dynamics using the RSVP may represent a valuable support tool in the context of AD screening. Furthermore, the RSVP is a computerized, auto-scored, and potentially self-administered cognitive test, which could be implemented as an online service to maximize accessibility. In future studies, we plan to administer the RSVP through a web application, and to validate the preliminary results obtained in this work on a larger participant sample.

## Data Availability

The dataset analyzed in this work is available upon request from the corresponding author.

## Abbreviations

AD: Alzheimer’s disease
MCI: Mild cognitive impairment
CDT: Clock-drawing test
RSVP: Rapid serial visual presentation
AB: Attentional blink
AM: Attentional masking
CBNU: Cognitive and behavioral neurology unit
CI: Cognitive impairment group
HC: Healthy control group
CSF: Cerebrospinal fluid
PET: Positron emission tomography
FBB: Florbetaben
LR: Logistic regression

## References

[1] Knopman DS, Petersen RC (2014). Mild cognitive impairment and mild dementia: a clinical perspective. Mayo Clin Proc.

[2] World Alzheimer Report 2022: Life after diagnosis: Navigating treatment, care and support (2022). Accessed 2023-02-23.

[3] Minati L, Edginton T, Grazia Bruzzone M, Giaccone G (2009). Reviews: current concepts in Alzheimer’s disease: a multidisciplinary review. Am J Alzheimer’s Dis Other Dementias.

[4] Perrin RJ, Fagan AM, Holtzman DM (2009). Multimodal techniques for diagnosis and prognosis of Alzheimer’s disease. Nature..

[5] Petersen RC, Doody R, Kurz A, Mohs RC, Morris JC, Rabins PV, Ritchie K, Rossor M, Thal L, Winblad B (2001). Current concepts in mild cognitive impairment. Arch Neurol.

[6] Meek PD, McKeithan EK, Schumock GT (1998). Economic considerations in Alzheimer’s disease. Pharmacotherapy.

[7] Raza M, Awais M, Ellahi W, Aslam N, Nguyen HX, Le-Minh H (2019). Diagnosis and monitoring of Alzheimer’s patients using classical and deep learning techniques. Expert Syst Appl.

[8] Lu D, Popuri K, Ding GW, Balachandar R, Beg MF (2018). Multiscale deep neural network based analysis of FDG-PET images for the early diagnosis of Alzheimer’s disease. Med Image Anal.

[9] Palmqvist S, Zetterberg H, Mattsson N, Johansson P, For the Alzheimer’s Disease Neuroimaging Initiative, Minthon L, Blennow K, Olsson M, For the Swedish BioFINDER study group, Hansson O. Detailed comparison of amyloid PET and CSF biomarkers for identifying early Alzheimer disease. Neurology. 2015;85(14):1240–9. 10.1212/WNL.0000000000001991. 10.1212/WNL.0000000000001991 PMC4607601 26354982

[10] Breton A, Casey D, Arnaoutoglou NA (2019). Cognitive tests for the detection of mild cognitive impairment (MCI), the prodromal stage of dementia: meta-analysis of diagnostic accuracy studies. Int J Geriatric Psychiatry.

[11] Hogervorst E, Combrinck M, Lapuerta P, Rue J, Swales K, Budge M (2002). The Hopkins verbal learning test and screening for dementia. Dement Geriatr Cogn Disord.

[12] Carnero-Pardo C, Sáez-Zea C, Montiel-Navarro L, Feria-Vilar I, Gurpegui M (2011). Estudio normativo y de fiabilidad del fototest. Neurologia.

[13] Perez-Valero E, Lopez-Gordo MA, Morillas C, Pelayo F, Vaquero-Blasco MA (2021). A review of automated techniques for assisting the early detection of Alzheimer’s disease with a focus on EEG. J Alzheimer’s Dis: JAD.

[14] Matías-Guiu JA, Valles-Salgado M, Rognoni T, Hamre-Gil F, Moreno-Ramos T, Matías-Guiu J (2017). Comparative diagnostic accuracy of the ACE-III, MIS, MMSE, MoCA, and RUDAS for screening of Alzheimer disease. Dement Geriatr Cogn Disord.

[15] del Ser T, Sánchez-Sánchez F, Garcíade Yébenes MJ, Otero A, Munoz DG (2006). Validation of the seven-minute screen neurocognitive battery for the diagnosis of dementia in a Spanish population-based sample. Dement Geriatr Cogn Disord.

[16] Tombaugh TN, McIntyre NJ (1992). The mini-mental state examination: a comprehensive review. J Am Geriatr Soc.

[17] Nasreddine ZS, Phillips NA, Bédirian V, Charbonneau S, Whitehead V, Collin I, Cummings JL, Chertkow H (2005). The Montreal Cognitive Assessment, MoCA: a brief screening tool for mild cognitive impairment. J Am Geriatr Soc.

[18] Senior K (2009). Test your memory–a waiting room test for dementia. Nat Rev Neurol.

[19] Carnero-Pardo C, Cruz-Orduña I, Espejo-Martínez B, Martos-Aparicio C, López-Alcalde S, Olazarán J. Utility of the mini-cog for detection of cognitive impairment in primary care: data from two spanish studies. Int J Alzheimer’s Dis. 2013;2013. 10.1155/2013/285462 PMC3771448 24069544

[20] Pinto E, Peters R (2009). Literature review of the clock drawing test as a tool for cognitive screening. Dement Geriatr Cogn Disord..

[21] Davis C, Heidler-Gary J, Gottesman RF, Crinion J, Newhart M, Moghekar A, Soloman D, Rigamonti D, Cloutman L, Hillis AE (2010). Action versus animal naming fluency in subcortical dementia, frontal dementias, and Alzheimer’s disease. Neurocase.

[22] Hodkinson HM (1972). Evaluation of a mental test score for assessment of mental impairment in the elderly. Age Ageing.

[23] Bublak P, Redel P, Sorg C, Kurz A, Förstl H, Müller HJ, Schneider WX, Finke K (2011). Staged decline of visual processing capacity in mild cognitive impairment and Alzheimer’s disease. Neurobiol Aging.

[24] Khaligh-Razavi S-M, Habibi S, Sadeghi M, Marefat H, Khanbagi M, Nabavi SM, Sadeghi E, Kalafatis C (2019). Integrated cognitive assessment: speed and accuracy of visual processing as a reliable proxy to cognitive performance. Sci Rep.

[25] Kavcic V, Duffy CJ (2003). Attentional dynamics and visual perception: mechanisms of spatial disorientation in Alzheimer’s disease. Brain J Neurol.

[26] Peters F, Ergis A-M, Gauthier S, Dieudonné B, Verny M, Jolicoeur P, Belleville S (2012). Abnormal temporal dynamics of visual attention in Alzheimer’s disease and in dementia with Lewy bodies. Neurobiol Aging.

[27] Park J, Jeong E, Seomun G (2018). The clock drawing test: a systematic review and meta-analysis of diagnostic accuracy. J Adv Nurs.

[28] Amodeo S, Mainland BJ, Herrmann N, Shulman KI (2015). The times they are a-changin’: clock drawing and prediction of dementia. J Geriatr Psychiatry Neurol.

[29] Souillard-Mandar W, Davis R, Rudin C, Au R, Libon DJ, Swenson R, Price CC, Lamar M, Penney DL (2016). Learning classification models of cognitive conditions from subtle behaviors in the digital Clock Drawing Test. Mach Learn.

[30] Olazarán J, Hoyos-Alonso MC, del Ser T, Garrido Barral A, Conde-Sala JL, Bermejo-Pareja F, López-Pousa S, Pérez-Martínez D, Villarejo-Galende A, Cacho J, Navarro E, Oliveros-Cid A, Peña-Casanova J, Carnero-Pardo C (2016). Aplicación práctica de los test cognitivos breves. Neurologia.

[31] Carnero-Pardo C, Espejo-Martínez B, López-Alcalde S, Espinosa-García M, Sáez-Zea C, Hernández-Torres E, Navarro-Espigares JL, Vílchez-Carrillo R (2011). Diagnostic accuracy, effectiveness and cost for cognitive impairment and dementia screening of three short cognitive tests applicable to illiterates. PLOS ONE.

[32] Carnero-Pardo C, Lopez-Alcalde S, Allegri RF, Russo MJ (2014). A systematic review and meta-analysis of the diagnostic accuracy of the Phototest for cognitive impairment and dementia. Dement Neuropsychol..

[33] Carnero-Pardo C, Sáez-Zea C, Montiel-Navarro L, Feria-Vilar I, Gurpegui M (2011). Normative and reliability study of fototest. Neurología (English Edition).

[34] Navarro C, Muñoz I, Cueva L, López-Alcalde S, García M, Carnero-Pardo C (2015). Evaluation of the equivalence of three parallel versions of the Phototest. Alzheimer.

[35] McGraw KO, Wong SP (1992). A common language effect size statistic. Psychol Bull.

[36] Carnero-Pardo C, Espejo-Martinez B, Lopez-Alcalde S, Espinosa-Garcia M, Saez-Zea C, Vilchez-Carrillo R, Hernandez-Torres E, Navarro-Espigares JL (2011). Effectiveness and costs of phototest in dementia and cognitive impairment screening. BMC Neurol.

[37] Powlishta KK, Dras DDV, Stanford A, Carr DB, Tsering C, Miller JP, Morris JC (2002). The clock drawing test is a poor screen for very mild dementia. Neurology..

[38] Russo MJ, Iturry M, Sraka MA, Bartoloni L, Carnero Pardo C, Allegri RF (2014). Diagnostic accuracy of the Phototest for cognitive impairment and dementia in Argentina. Clin Neuropsychol.

[39] Ruiz-Rizzo AL, Bublak P, Redel P, Grimmer T, Müller HJ, Sorg C, Finke K (2017). Simultaneous object perception deficits are related to reduced visual processing speed in amnestic mild cognitive impairment. Neurobiol Aging.

[40] Salobrar-García E, Hoz RD, Ramírez AI, López-Cuenca I, Rojas P, Vazirani R, Amarante C, Yubero R, Gil P, Pinazo-Durán MD, Salazar JJ, Ramírez JM (2019). Changes in visual function and retinal structure in the progression of Alzheimer’s disease. PLOS ONE.

[41] Almario G, Piñero DP (2021). Impact of Alzheimer’s disease in ocular motility and visual perception: a narrative review. Semin Ophthalmol..

[42] Chang C-W, Su K-C, Lu F-C, Cheng H-M, Cheng C-Y (2022). Visual function and visual perception among senior citizens with mild cognitive impairment in Taiwan. Healthcare..

[43] Krajcovicova L, Barton M, Elfmarkova-Nemcova N, Mikl M, Marecek R, Rektorova I (2017). Changes in connectivity of the posterior default network node during visual processing in mild cognitive impairment: staged decline between normal aging and Alzheimer’s disease. J Neural Transm.

[44] Timothy LT, Krishna BM, Nair U (2019). Recurrence quantification analysis of mci eeg under resting and visual memory task conditions. Biomed Eng Appl Basis Commun..

[45] Hashemi A, Roudaia E, Anderson ND, Alain C, Aleong R, Khatri N, Freedman M, Sekuler AB (2020). Behavioural and electrophysiological measures of visual processing for early detection of Alzheimer’s disease. J Vis.

[46] Yamasaki T (2021). Use of VEPs as electrodiagnostic biomarkers of mild cognitive impairment. Neurol Clin Neurosci.

[47] Ye S, Sun K, Huynh D, Phi HQ, Ko B, Huang B, Ghomi RH (2020). Validation of a computerized cognitive test battery for detection of dementia and mild cognitive impairment. Neurology.

[48] Thabtah F, Peebles D, Retzler J, Hathurusingha C (2020). A review of dementia screening tools based on Mobile application. Heal Technol.

[49] Perin S, Buckley RF, Pase MP, Yassi N, Lavale A, Wilson PH, Schembri A, Maruff P, Lim YY (2020). Unsupervised assessment of cognition in the Healthy Brain Project: implications for web-based registries of individuals at risk for Alzheimer’s disease. Alzheimer’s Dement.

[50] Visser LNC, Dubbelman MA, Verrijp M, Wanders L, Pelt S, Zwan MD, Thijssen DHJ, Wouters H, Sikkes SAM, Hout HP, van der Flier WM (2021). At-home assessment of cognitive performance: establishing norm scores for the Cognitive Online Self-Test Amsterdam (COST-A). Alzheimer’s Dement.

[51] Buitinck L, Louppe G, Blondel M, Pedregosa F, Mueller A, Grisel O, Niculae V, Prettenhofer P, Gramfort A, Grobler J, Layton R, Vanderplas J, Joly A, Holt B, Varoquaux G. API design for machine learning software: experiences from the scikit-learn project. arXiv. arXiv:1309.0238 [cs] (2013). 10.48550/arXiv.1309.0238. Accessed 2023-02-23.

